# Middle-East OBGYN Graduate Education (MOGGE) Foundation Practice Guidelines: Prelabor rupture of membranes; Practice guideline No. 01-O-19

**DOI:** 10.7189/jogh.10.010325

**Published:** 2020-06

**Authors:** Sherif A Shazly, Islam A Ahmed, Ahmad A Radwan, Ahmed Y Abd-Elkariem, Nermeen Bahaa El-Dien, Esraa Y Ragab, Mostafa H Abouzeid, Ahmed H Shams, Ahmed K Ali, Heba N Hemdan, Menna N Hemdan, Ahmed A Nassr, Faten F AbdelHafez, Nashwa A Eltaweel, Khaled Ghoniem, Ali M El Saman, Mohamed K Ali, Angela C Thompson, Middle-East Obstetrics and Gynecology Graduate Education (MOGGE) Foundation Practice Group

**Affiliations:** 1 Department of Obstetrics and Gynecology, Mayo Clinic, Rochester, Minnesota, USA; 2 Department of Obstetrics and Gynecology, Assiut School of Medicine, Assiut, Egypt; 3 Faculty of Medicine, South Valley University, Egypt; 4 Department of Obstetrics & Gynecology, Baylor College of Medicine and Texas Children's Hospital, Houston, Texas, USA; 5 York Teaching Hospital NHS Foundation Trust, York City, UK

Prelabor rupture of membranes (PROM) refers to rupture of membranes prior to the onset of labor. PROM is a common obstetric disorder that may be associated with significant maternal, fetal and neonatal complications. Diagnosis and management of PROM have been thoroughly investigated in the literature. Nevertheless, many decisions are still debatable. Practice in low-resource settings is prone to several challenges including resource, training, and awareness barriers. This article provides review of the literature and analysis of evidence of management of PROM in general, with attention to debates particularly in low-resource settings.

**Figure:**
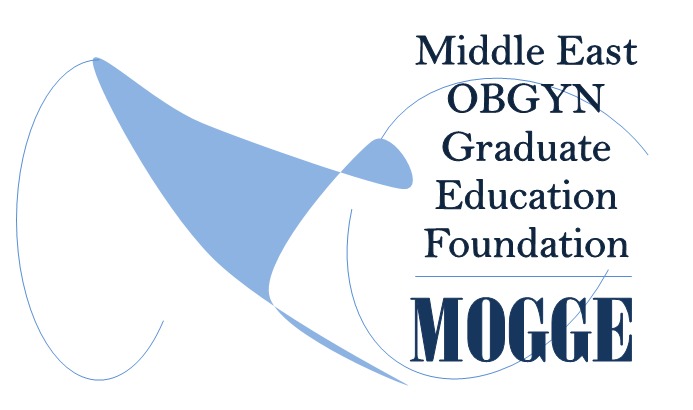
Photo: The Middle East OBGYN Graduate Education Foundation.

## INTRODUCTION | KNOWLEDGE

### Definitions

Although the classic term “premature rupture of membranes” has been recently modified to “prelabor rupture of membranes”, the abbreviation (PROM) as well as the definition of the condition have not been changed and are satisfactorily consistent worldwide. The new term “prelabor rupture of membranes” has been adopted by the American College of Obstetricians and Gynecologists (ACOG) in 2018 and even earlier by the National Institute of Clinical Excellence (NICE) [1,2]. We believe that the new term is more accurate and should be widely used instead of the old term. Here, the old term “premature” may be misinterpreted as “fetal prematurity” and the use of both “preterm” and “premature” to define the condition is confusing. Therefore, we highly recommend the use of the new term instead of the old one. Prelabor rupture of membranes is classified according to the gestational age at which it occurs into; term, preterm and previable:

- *Term pre-labor rupture of membranes:* Rupture of the membranes at or beyond 37 weeks’ gestation prior to the onset of labor. This definition is consistent with ACOG practice bulletins and the Royal Australian and New Zealand College of Obstetricians and Gynaecologists (RANZCOG) guidelines [1,3].
- *Preterm pre-labor rupture of membranes (PPROM):* Rupture of membranes prior to 37 weeks of gestation and before the onset of labor. This is opposed to rupture of membranes during actual preterm labor. The definition is similarly defined by ACOG, NICE, RANZCOG and World Health Organization (WHO) [1-4].
- *Previable pre-labor rupture of membranes (previable PROM):* as defined by the ACOG, rupture of membranes that occurs before the gestational age of viability [1]. This category should be separated from PPROM because counseling and management are significantly different. Although the definition of previable PROM is universally acceptable, the definition of viability changes over time and it varies widely between low and high resource countries.

According to Websters Encyclopedic Unabridged Dictionary, the word “viability” refers to the stage of fetal development at which a fetus is capable of living outside the uterus [5]. Clinically, the age of viability is defined as the gestational age at which a fetus has a 50% chance to survive after delivery [6]. The age of viability is inconsistent worldwide as survival of the premature fetus is highly dependent on immediate neonatal care. Biomedical maturity and technological advancement are the determinants of viability [7]. Therefore, gestational age at viability in the Middle-East, Africa and other low resource countries is generally greater than that in developed countries [5]. In the United States, the limit of viability is 24 weeks’ gestation because of advanced neonatal care. Moreover, some institutes set the definition to 23 weeks’ gestation based on their internal data [8]. In Portugal, the limit of viability ranges between 25 and 26 weeks’ gestation [9]. In India, an Asian country, neonatal mortality is 55% when delivered at 26 to 27 weeks’ gestation [10]. On the other side, African countries like Nigeria typically set there limit of viability to 28 weeks’ gestation [11]. Although there is insufficient data, 28 weeks seems to be the most acceptable cut-off among obstetricians in the Middle-East.

### Regional challenges

Although diagnosis and management of PROM has been relatively consistent among internationally recognized obstetric committees, the practice in low-resource settings is typically challenged by several issues that need to be addressed. As mentioned before, **gestational age at viability** is a determinant of management plan and unfortunately, it significantly differs from viability limits in the developed countries. Furthermore, due to neonatal care advancement, neonatal outcomes are more favorable if delivered in high resource countries compared to resource challenged. Therefore, even newborns delivered at 34 weeks’ gestation do not necessarily present reassuring outcomes in low-resource settings. **Lack of excellent dating** presents another challenge that complicates the decision-making process.

Low resource settings may not have **access to some tests due to their cost**. Fortunately, current standard PROM management does not warrant expensive work-up. On the other side, other tests tend to be less expensive and easier to access in Middle-Eastern and African countries eg, obstetric ultrasound is done by the obstetrician as a part of prenatal visit with no or minimal extra-cost. Unfortunately, some non-expensive approaches are not commonly performed in these countries due to **lack of either awareness and/or experience** on their rule or technique (such as microscopic examination, ferning test,. etc).

Another challenge is related to **availability and proximity of tertiary care** in low-resource countries. In many areas, tertiary maternal care or neonatal intensive care may be more than 150 miles away from residential areas. Given the fact that air transportation is not typically an option, attention to these constraints should be paid to ensure patient safety and guide clinical management at location of presentation.

### Resources and adoption approach

Many aspects of management of PROM have been adequately addressed either by internationally recognized guidelines or by local practice policies. Our approach starts with **identifying clinical questions** that need to be answered, achieved by thorough reviewing of the latest versions of high-quality guidelines that are representative of global practice. Most recent versions of published guidelines were considered (to February 2019). These guidelines include WHO recommendations (November 2015) [4], ACOG practice bulletin (January 2018) [1], the Society of Obstetricians and Gynecologists of Canada (SOGC) (September 2017) [12], the RANZCOG (March 2017), and NICE guidelines (November 2015) [2,3]. The second step, each clinical question is assigned to a panel of 2 researchers to **review how a question is covered in these guidelines.** The aim is to verify if a question is consistently answered, to identify the range of answers provided if inconsistent among different guidelines, and to assess the adequacy of evidence supporting these answers. The third step is **to search the literature for newly published studies** related to the topic as well as the original studies conducted primarily in the Middle-East or alternatively, in low resource countries that may share similar challenges. MEDLINE, EMBASE, SCOPUS and Cochrane library were searched for prospective studies, clinical trials, systematic reviews and meta-analyses from 2000 to June 2019. Search terms include “rupture of membranes”, “PROM”, “PPROM”, “previable”, “induction of labor”, “chorioamnionitis”, “latency antibiotics”, “antenatal steroids”, and “preterm labor”. The fourth step is to **survey obstetricians in the Middle-East** to identify their needs, challenges and actual practice. These surveys include obstetricians at different levels of experience, who are involved in either community based or university-based practice. The fifth and final step is to **review the outcomes of these steps and fit the highest level of evidence** that is appropriate for low resource settings.

The level of evidence is determined in accordance to Oxford Centre for Evidence-based Medicine – Levels of Evidence, which stratifies studies depending on their design to level 1 (A to C), level 2 (A to C), level 3 (A and B), level 4, and level 5 [13] (Appendix1 in the **Online Supplementary Document**). A KAST approach will be used to present this topic; Knowledge, Assessment, Sharing decision, and Treatment (**Table 1**).

**Table 1:** MOGGE take-home message: Knowledge

• The term “prelabor rupture of membranes” is more universally acceptable than “premature rupture of membranes”
• “Preterm prelabor rupture of membranes” corresponds to prelabor rupture of membranes prior to 37 weeks’ gestation
• Gestational age before which the diagnosis of “Previable prelabor rupture of membranes is made is inconsistent. Age of viability is determined by neonatal outcomes per each facility

MOGGE – Middle-East OBGYN Graduate Education

## CLINICAL EVALUATION AND DIAGNOSIS | ASSESSMENT

### Initial assessment

Clinical assessment aims to confirm the diagnosis and minimize introduction of infection. **History taking** should include; time of onset of fluid leakage, description of the amount and color of leaking fluid, onset of uterine contractions (if present) in relation to fluid leakage, and the presence of abdominal pain or vaginal bleeding. History alone does not confirm or rule out the diagnosis and thus a thorough **physical examination** is essential in all cases.

It starts with assessment of vital signs, of which temperature is particularly important because of the risk of intra-amoniotic infection (IAI). Abdominal examination is performed to rule out abdominal tenderness that may indicate chorioamnionitis or placental abruption, to palpate any uterine contractions and to assess the fetal presentation. **Sterile speculum examination** is used to visualize the cervix. Diagnostic signs of PROM during speculum examination includes a direct leakage from the cervix and “pooling” of fluid at the posterior fornix. Valsava maneuver may be attempted if no fluid leakage can be appreciated. Speculum examination may also detect umbilical cord prolapse, cervical dilation, or vaginal bleeding. Vaginal swab should be obtained for culture if infection is suspected. Speculum examination can be performed shortly after presentation if there is subjective or objective evidence of ongoing leakage or if there is any clinical concern that warrants immediate assessment. Otherwise, the patient may be given a 30-minute period in a semi-Fowlers position to allow “pooling” of amniotic fluid in the vagina. Placement of a sterile pad for 30 minutes to 1 hour can also help to identify the color and odor of the leaking fluid. This can differentiate amniotic fluid from urine, which seems to be the most common differential diagnosis in these cases [4].

Digital examination is not superior to a speculum inspection and it should be avoided to reduce the risk of infection [14]. Digital examination may be warranted if the patient has apparently frequent and efficient contractions. However, if induction of labor is planned and no uterine contractions are appreciated, initiation of induction without a digital examination is justified. As a rule, digital examination is to be avoided unless it may alter the clinical decision.

This clinical approach is supported by ACOG, NICE and RANZCOG and is appropriate for low-resource settings [1-3]. Because of low-resource practice challenges including the possibility of delayed presentation, we would like to emphasize the WHO recommendations to further reduce the risk of infection [4]. In addition to the use of a sterile speculum, attention should be paid to hand washing and using gloves. On the other side, prevention of infection should extend to physicians who should handle any vaginal discharge including blood with caution [4], and universal precautions to prevent infection transmission is advised and is standard of care.

### Confirmation of diagnosis and work-up

Diagnosis is made following history and physical examination in most cases [6]. Visualization of **fluid pooling** in the posterior cervix is the basis of diagnosis. However, several tests are available to confirm that it is amniotic fluid. These tests include “**ferning**” of dried vaginal fluid under microscopic examination, **basic vaginal fluid pH** (Nitrazine test), and the use of **amniotic fluid detection strips**, if pooling is not visualized.

Both microscopic examination of ferning and vaginal pH testing require some degree of fluid pooling to allow proper testing and interpretation. Microscopic examination is performed by collecting pooled vaginal fluid and smearing it over a clean microscope slide, the specimen is left to air dry without covering. Long standing PROM with absence of ongoing leakage may lead to false-negative results for both tests. For microscopic examination, heavy contamination with blood or vaginal discharge is another cause of false-negative results. Fingerprints on the slide may result in false-positive results. Overall, sensitivity and specificity of microscopic examination are 98% and 88.2% among laboring women, 51.3% and 70.5% among non-laboring women, respectively [15]. On the other hand, a basic vaginal pH is not specific; the presence of blood, semen or bacterial vaginosis, may produce false-positive results [16]. Therefore, Nitrazine test is not supported by NICE recommendations [2].

In low-resource settings, we highly recommend the implementation of these relatively inexpensive tests, which facilitates correct diagnosis and management of this common problem and hence, minimizes diagnostic errors. Given the fact that vaginal pH testing may be misled by false-positive result and the need for supplies, we recommend microscopic examination over Nitrazine test in low resource settings. Despite its simplicity, when 597 obstetricians from the Middle-East were surveyed whether ferning test is implemented in their practice; 575 (96.3%) reported that they did not endorse it as a part of their practice. Therefore, we believe implementation of this test should be further supported in low resource setting knowing that this test is supportive and that a negative test does not exclude diagnosis particularly in non-laboring women.

In the absence of pooling, commercially available detection kits can identify amniotic fluid-specific proteins in vaginal fluid, specifically growth factor binding protein-1 test (AmnioQuick Duo+, Biosynex, Strasbourg, France) and placental alpha-microglobulin-1 test (Amnisure®, Qiagen N.V., Venlo, the Netherlands]). A recent multicenter prospective cohort study on 99 women concluded that both tests are comparable in diagnostic accuracy [16]. These tests have high negative predictive value for exclusion of PROM. Nevertheless, they yield high false-positive results among term and preterm women (31% and 19%, respectively) [17,18]. Another test, called ROM Plus® (Clinical Innovations, Salt Lake City, UT USA), is designed to detect both placental protein 12 and alpha-fetoprotein as indicators of amniotic fluid. Compared to Amnisure, a prospective study on 111 women showed comparability in diagnostic accuracy [19]. These tests seem to have limited confirmatory role and are not only expensive but also unavailable at many low-resource settings. Therefore, their implementation is acceptable but not essential given their potential cost-ineffectiveness. And risk of false-positive results would be especially risky in lower resources areas if delivery is recommended at early fetal gestation.

**Bed-side ultrasound** is an essential part of clinical assessment, primarily to confirm fetal presentation. Although clinical diagnosis of fetal presentation, using Leopold maneuvers, is a common practice in low-resource settings, a cross-sectional study on 1633 women with a singleton pregnancy between 35 and 37 weeks' gestation showed that sensitivity of clinical examination for diagnosis of non-cephalic presentation is not ideal (70%) [20]. Because fetal presentation significantly impacts the decision-making process for delivery, a screening for fetal presentation is necessary regardless of abdominal examination. **Assessment of amniotic fluid volume** may be a useful adjuvant test if the diagnosis is not definite. However, absence of oligohydramnios should not be used to rule out diagnosis. On the other side, amniotic fluid index (AFI) is commonly used in the Middle East, which can lead to over-diagnosis of oligohydramnios. A Cochrane review that included 5 clinical trials in a meta-analysis, concluded that the use of maximum vertical pocket (MVP) is superior to AFI, as the later increases the rate of diagnosis of assumed oligohydramnios, without improving perinatal outcomes [21]. A recent clinical trial (SAFE trial) has supported the same conclusions among low-risk women [22]. Because amniotic fluid volume is not diagnostic, there is no adequate data whether AFI is more informative than maximum vertical pocket. Therefore, the later should still be used to indicate oligohydramnios.

**Although trans-vaginal cervical length assessment** is a part of preterm labor work-up because of its predictive value, its rule in PROM is doubtful. A prospective study on 101 women with PROM suggested a predictive value of transvaginal measurement of cervical length for early labor but not for chorioamnionitis [23]. Another prospective study on 55 women with PPROM evaluated the use of trans-labial technique and did not find cervical length informative on either latency or risk of infection [24].

If the diagnosis is still unclear, the **ultrasound guided trans-abdominal instillation of indigo carmine dye** is typically performed if all other tests are equivocal. A positive test is indicated by blue staining of a vaginal tampon or pad [6]. Although the procedure is diagnostic, it is invasive, and should be performed only by a physician who is expert in performing it. Therefore, conduction of such a test in low-resource settings may be challenging and should not be implemented unless feasibility and safety are ensured. Initial assessment of fetal status is an essential part of management planning. Electronic fetal heart rate monitoring with monitoring of uterine activity is warranted at the time of presentation. No laboratory tests are required if the mother and the fetus are stable [1], and no signs and symptoms of IAI present (**Table 2**).

**Table 2:** MOGGE take-home message: Assessment

• History taking is an important part in the diagnosis of PROM. However, physical examination is necessary to confirm the diagnosis
• Initial assessment should include; evaluation of maternal vital signs, fetal heart rate monitoring, abdominal examination and sterile speculum examination
• Unless immediate assessment is warranted, examination can be shortly postponed allowing for the pooling of fluid in the vagina
• Vaginal “pooling” of amniotic fluid and microscopic examination positive for ferning are sufficient for diagnosis
• “Nitrazine” test, amniotic fluid detection kits, and instillation of indigo carmine dye. ‎are less recommended for assessment of Preterm PROM
• Clinical assessment should not be used alone for determination of fetal presentation
• Assessment of maximum vertical pocket is recommended over AFI for diagnosis of oligohydramnios
• Cervical length assessment is not indicated in women with Preterm PROM

MOGGE – Middle-East OBGYN Graduate Education. PROM – prelabor rupture of membranes, AFI – amniotic fluid index

## DECISION MAKING AND PATIENT COUNSELING | SHARING DECISION

### Current pregnancy prognosis

PROM has a range of maternal, fetal and neonatal outcomes. It ranges from spontaneous sealing of the defect – being the most favorable outcome – to major maternal and fetal morbidity or mortality as the worst prognosis [25]. One of our priorities in this article is to reinforce the value of patient counseling and shared decision making in high volume low resource settings where this process can, sometimes, be deficient. We believe that after assessment is complete, potential management plan and prognosis should be discussed with the patient. It is important to involve the partner and other members of the family if the patient prefers them to be involved. Counseling should include anticipated maternal, fetal and neonatal outcomes, potential complications including the plan to prevent or diagnose them early, precautions for patient, and management plan. A period is given for the patient and the family to ask questions. Eventually, the patient either agrees to the treatment plan or rejects any part of it as long as her decision is made on clear understanding of the risks and benefits. Documentation should be a part of standard practice. Given the fact that availability of resources is limited in these settings especially human resources, we highly recommend the use of visual aids (eg, pamphlets) for patient education. Also, we recommend regular training for nurses to be able to respond to patients` basic questions and concerns. Overall, low-resource settings should pay more attention to nurse involvement in patient care process, being the first line of contact with the patient.

The process of counseling and decision making should be individualized based on several factors. The following 5 points should be discussed with the patient.

#### I. Expected outcomes according to gestational age (previable, preterm or term PROM)

PROM neonatal complications include respiratory distress syndrome, intraventricular hemorrhage, sepsis and necrotizing enterocolitis [26]. Gestational age is the most significant determinant of these complications.

##### 1. Previable prelabor rupture of membrane

Even at high-resource settings, rate of perinatal death when PROM occurs prior to week 22 is approximately 58% and is primarily attributed to pulmonary hypoplasia. Alveolar development takes place around 23 weeks of gestation and the rate of perinatal death declines significantly beyond 24 weeks of gestation [27,28], probably as alveolar function becomes mature enough to perform respiration outside the uterus [29,30]. Therefore, age of viability among high-resource settings ranges between 23-24 weeks of gestation. Nevertheless, neonatal survival at this gestational age requires intensive neonatal care and resources that are not available in most low-resource settings. Favorable outcomes are less likely before 28 weeks of gestation in low resource settings. Therefore, patient counseling should be guided by local neonatal data. We surveyed 370 obstetricians on gestational age at viability based on their practice and internal perinatal data, it was defined as 28 weeks by 310 obstetricians (83.8%), 24 weeks by 32 obstetricians (8.6%), 29 weeks by 24 obstetricians (6.5%), and 26 weeks by 4 obstetricians (1.1%).

It is important to discuss PROM at a previable gestational age is associated with pulmonary hypoplasia secondary to prolonged oligohydramnios. Oligohydramnios also results in limb contractures and potter like deformities (low set ears and epicanthus folds). However, skeletal positioning deformities likely resolve by physiotherapy [27,31]. Maternal risks should also be reviewed thoroughly. Maternal risk of infection, which may evolve into life-threatening sepsis, can occur in 1%.

In summary, previable PROM should be discussed as to provide realistic expectations. Involvement of an obstetrician and a neonatologist is highly recommended to optimize this discussion.

##### 2. Preterm prelabor rupture of membrane

Patient should be counseled that conservative management is the standard approach if both the mother and the fetus are stable. However, she should be aware of possible maternal/fetal complications and their incidence, because some of these complications are common, serious, and may develop abruptly. These complications include ascending infection (15%-25%) [32], postpartum infection (15%-20%) [33,34], placental abruption (2%-5%) [35,36], and antenatal fetal demise secondary to umbilical cord accident (1%-2%) [37]. Although the latter is not common, it cannot be predicted by antenatal fetal surveillance. In addition, complications of prematurity apply to this gestational age and the patient should be aware that lack of immediate neonatal intervention significantly worsens outcomes eg, spontaneous delivery before safe transportation to an equipped facility. Explanation of these complications is important to justify the risks of home management as well as prolonged expectant management beyond 34 weeks of gestation.

##### 3. Term prelabor rupture of membrane

Term PROM is common (8%) and is associated with the best prognosis. Counseling should emphasize the necessity of immediate delivery given the previously mentioned complications that may develop with expectant management. In this situation, benefits of expectant management do not outweigh the risks. There is also a much latency period after term PROM with labor occurring spontaneously within 24 hours of membrane rupture at term [38]. However, shared decision should take into consideration transfer to a tertiary care center and local neonatal outcome data per gestational age, should there be a question regarding optimal dating of gestational age.

#### II. Expected latency between PROM and delivery

Latency is defined as the time interval between prelabor rupture of membranes and onset of spontaneous delivery. As a role, latency is inversely correlated with gestational age. For instance, the incidences of spontaneous labor at 24, 48 and 96 hours after term PROM are 70%, 85%, and 95%, respectively [38,39], while 50% of women with preterm PROM deliver within 168 hours [40]. Nevertheless, the exact latency cannot be accurately predicted.

#### III. Active measurements to minimize neonatal infection

PROM is a condition where the natural barrier between the fetus and the pool of microorganisms in the lower genital tract is broken. Nevertheless, prolonged prophylactic antibiotic courses are not universally recommended because the risk of drug resistance overweighs the benefits of prophylaxis. The status of 3 microorganisms should be documented when a patient is recently diagnosed with PROM prior to plan for care: Group B streptococci (GBS), human immunodeficiency virus (HIV) and herpes simplex virus (HSV). These infections can be transmitted through the birth canal to the fetus. For GBS, a rectovaginal swab should be obtained and sent for culture. Sensitivity may be indicated if the patient is allergic to Penicillin. Women at risk of genital HSV or has prior history of genital HSV should undergo a speculum examination to rule out active lesions at the time of presentation when they can be treated and at the onset of labor to decide whether to deliver vaginally or via a cesarean section. A positive HIV status warrants treatment during pregnancy. HIV viral load should be tested at the time of labor to decide if vaginal delivery is safe for the newborn [3]. This information should be shared with the patient.

Although the prevalence of HIV among Middle East countries remains low, approximately <1% of the population, it should be noticed that newly diagnosed cases have increased by 31% between 2001 and 2015 [3]. Therefore, we recommend that obstetric providers do not forego HIV testing among pregnant women in these countries.

#### IV. Hospitalization vs home-based care

It is not uncommon among population served by low-resource setting and among Middle-Eastern population that home-based care is requested. Currently, ACOG does not support home-based care because there is no sufficient evidence to confirm safety [1]. A Cochrane review was published on 2014 based on data derived from 2 clinical trials with a total of 116 women, with preterm PROM, and did not show significant difference in morbidity and mortality between the 2 groups. Women who were managed at hospital were more likely to deliver via cesarean section while women who were managed at home were more satisfied. However, the size of these studies is still small to conclude robust recommendations. In addition, women who are candidates for home management should fit strict criteria; patient should be reliable to watch for signs of infection or abruption, and should live close to a tertiary care hospital with dependable transportation [3]. Home management of term PROM seems to be less reasonable and is associated with higher risk of maternal need for antibiotics (odds ratio OR 1.52) and neonatal infection (OR 1.97) [41].

Therefore, we recommend that women with preterm PROM be hospitalized. Home care should not be offered as an alternative, given the limited supportive evidence. It can still be considered an option for women who refuse prolonged hospitalization if they meet the above criteria. However, thorough counseling is warranted to explain the risk of delayed management of PROM complications, to emphasize that abrupt onset of warning symptoms is not uncommon, and to highlight the paucity of studies that address the safety of home approach to management. Under these circumstances, home monitoring of temperature at least twice daily and twice weekly office visits should be discussed with the patient [2].

#### V. Impact of fetal presentation on neonatal prognosis

There is evidence that non-cephalic presentation may be associated with worse neonatal prognosis compared to cephalic presentation at the time of diagnosis. Secondary analysis of data from 1767 women from the reported Maternal-Fetal Medicine Units Network BEAM (Beneficial Effects of Antenatal Magnesium Sulfate; 1997-2004) trial who had preterm delivery revealed increased risk of neonatal death prior to dismissal if fetal presentation is non-cephalic. There was no associated increase in latency, risk of abruption, or neonatal morbidity [42]. Interpretation and counseling of these results should be carefully conducted as the mechanism is not clearly understood, and non-cephalic presentation should not justify external cephalic version (ECV); ECV among women with preterm PROM was associated with umbilical cord prolapse in one third of cases [43]. Moreover, there is no evidence that correction of fetal presentation would improve prognosis.

### Future pregnancy

History of PROM is a major risk for PROM in a subsequent pregnancy [44,45]. Although this risk factor is not modifiable, there are other **modifiable risk factors** that are associated with increased risk of PROM that may be discussed with the patient prior to her next pregnancy. Examples include short inter-pregnancy interval, low body mass index, cigarette smoking, and illicit drug use [46-50].

In addition, in case of previous preterm PROM, early intervention in the next pregnancy is more critical. **Cervical length** surveillance is widely supported by ACOG, NICE and WHO [1,2,4]. If PROM occurs prior to 34 weeks, the patient should be counseled that she can be offered **progesterone treatment** starting from 16 to 24 weeks' gestation with the possibility of cerclage placement if the cervical length is less than 25 mm prior to 24 weeks of gestation [51-53]. Intramuscular injection of 17-hydroxyprogesterone has been supported over vaginal progesterone by ACOG among women with prior preterm labor including prior PROM [1]. However, a recent meta-analysis of 3 RCTs with a total of 680 women with prior preterm labor concluded that daily vaginal progesterone (gel or suppositories) is an appropriate alternative to intramuscular progesterone. Unfortunately, quality of evidence of these studies is low and interpretation of these results should be taken cautiously [54]. Given the fact that intramuscular form may not be available, and administration of the medication warrants good patient complaint and availability of local nursing, we recommend considering daily vaginal progesterone as an alternative, if weekly intramuscular progesterone is not feasible.

Because management of future pregnancy is particularly important, improper **documentation** or lack of medical records in low resource settings can be a significant issue. Therefore, thorough history taking in first prenatal visit is mandatory. We highly recommend enforcing next pregnancy management discussion immediately after PROM delivery. Thereby, a patient is aware and is more proactive to bring this discussion to her provider in the next pregnancy and to bring a printed copy of her records if transfer of medical records may present an issue (**Table 3**).

**Table 3:** MOGGE take home message: Sharing decision

• Thorough counseling is essential after the diagnosis of PROM is made. Counseling should cover risks and justify plan of care
• Hospitalization remains the standard of care among women with preterm and term PROM. Home care should not be offered as an alternative due to limited evidence
• A provider should be aware of risks, home care selection criteria and warning signs to share with women who refuse hospitalization
• Although non-cephalic presentation may increase the risk of adverse neonatal outcomes, evidence is limited and ECV is not recommended
• After delivery, a patient should not be sent home without appropriate counseling on future pregnancy care. Precise documentation and patient education is highly recommended to avoid suboptimal care due to medical record transfer issues

MOGGE – Middle-East OBGYN Graduate Education. PROM – prelabor rupture of membranes, ECV – external cephalic version

## ACT: MANAGEMENT OF PROM | TREAT

### At 37 weeks of gestation or beyond

Once the diagnosis of term PROM is made, treatment plan should be discussed with the patient. As mentioned earlier, it is important to promote **hospitalization** regardless of labor status. Home management is not justifiable in this situation. Further management is influenced by maternal and fetal status, cervical dilation, fetal presentation, and presence of any indication for cesarean delivery.

If there is no contraindication to vaginal delivery and fetal status is reassuring, the goal is to **expedite vaginal delivery** if the patient is not in active labor on presentation.

Administration of **antibiotics** has been a practice issue in many countries in the world including many Middle-Eastern countries because prescription of antibiotics is not monitored, and they are available as over-the-counter medications. Laws and regulations are unlikely to change soon and therefore, it is the providers’ ethical and professional responsibility to limit antibiotic prescription and to enforce culture change among patients and younger physicians who may believe antibiotics are the first line of management. Drug resistance is a global threatening problem. Unfortunately, data from the Middle-East are sparse. However, a survey of antimicrobial susceptibility patterns from 5 hospitals in Cairo, Egypt, from 1999 to 2000 revealed high rates of resistance among most of the bacteria studied, specially gram-negative bacilli which were resistant to most relevant antibiotics [55].

Antibiotic administration is limited to 3 indications among women with term PROM: (1) prophylaxis against GBS; the indications are not any different from those conducted in uncomplicated labor; (2) Treatment of IAI if clinically suspected; if the patient experiences fever >39°C or persistently over 38°C along with leukocytosis, fetal tachycardia or purulent vaginal discharge according to ACOG committee opinion [1]. A key point to reduce the need for antibiotics is to minimize the number of digital pelvic examinations.

The third indication of antibiotic administration is prophylaxis against infection, and it presents the most controversial indication. A meta-analysis of 5 RCTs including 2699 women who were randomized to antibiotic group vs control group shows that antibiotic prophylaxis for term or near-term PROM is not associated with maternal or neonatal benefits. However, subgroup analysis of women with latency longer than 12 hours shows that women who received prophylactic antibiotics experience lower rates of chorioamnionitis and endometritis (risk ratio (RR) = 0.49, 95% CI = 0.27-0.91) and 0.12 (95% CI = 0.02-0.62), respectively [56]. Nevertheless, data from a local RCT conducted in Egypt in 2014 did not show statistical differences in the incidence of maternal infection or neonatal sepsis when prophylactic antibiotics were administered at or beyond 36 weeks of gestation [57]. We conducted a survey on 333 obstetricians; 291 (87.4%) stated that their practice supports prophylactic antibiotics for women with PROM from admission to delivery, 31 (9.3%) give antibiotics if latency exceeds 18 hours, 11 (3.3%) administer antibiotics only if GBS culture is positive or to treat chorioamnionitis.

Although the goal is to achieve expedited delivery, the decision whether to start induction of labor immediately or to consider initial watchful expectancy for possible spontaneous onset of labor is debatable among women who are not in active labor. Based on a Cochrane review of 23 RCTs in 2017, ACOG supports immediate induction of labor over expectant management because it reduces latency between PROM and delivery and thus, the risk of maternal and neonatal complications [1,58]. In women with intact membranes, cervical bishop score is used to assess cervical favorability and to determine whether intravenous oxytocin, misoprostol or Foley bulb is selected for induction of labor. Among women with term PROM, oxytocin may be superior to all other options. Among women with term PROM, induction of labor with oxytocin was associated with fewer digital vaginal examinations, shorter latent phase, shorter labor, and shorter hospital stay compared to prostaglandin induction [59]. Furthermore, application of vaginal prostaglandins can also be associated with the possibility of “washout”. On the other hand, the use of buccal prostaglandins may be associated with more systemic side effects including fever, which may interfere with monitoring of maternal infection. Placement of Foley bulb has been associated with theoretical concerns of chorioamnionitis risk due to the insertion of a foreign body. However, available evidence is limited. A retrospective study on 124 women showed increased risk of IAI with Foley bulb compared to oxytocin induction group. Yet, the risk is not statistically significant (*P* = 0.1) [60]. Preliminary results from Foley Bulb for Labor Induction in Premature Rupture of Membranes in Nulliparas (FLIP trial) which compared concurrent Foley bulb/oxytocin vs oxytocin alone did not show an increased risk of chorioamnionitis. Nevertheless, addition of a Foley bulb to oxytocin does not shorten induction to delivery time [61].

If cesarean delivery is indicated, preliminary results from a meta-analysis of 19 studies (including 6179 patients) undergoing cesarean delivery showed that vaginal irrigation with povidone-iodine 1% reduces the risk of endometritis and wound complications [62]. It is reasonable to consider this intervention given its simplicity and low cost, and increased risk of infection among women with PROM. Preterm breech presentation is an indication of cesarean delivery in the setting of PROM. ECV is an appropriate option in the absence of PROM. However, this procedure is associated with high risk of failure and significant maternal and fetal complications and is not recommended [63]. Although amnioinfusion may appear to be an option, a recent RCT (119 women) showed that it did not improve the success rates [64]. However, data on ECV following amnioinfusion in women with PROM are limited.

### Between 34-36 6/7 weeks of gestation

ACOG recommends immediate delivery once pregnancy is 34 weeks or beyond because conservative management is associated with an increased risk of amnionitis (16% vs 2%, *P* = 0.001), prolonged hospitalization (5.2 vs 2.6 days, *P* = 0.006), and lower mean umbilical cord pH at delivery (7.25 vs 7.35, *P* = 0.009) without improvement of prematurity related perinatal complications. More recent recommendations also support administration of a course of **antenatal steroids** between 34 0/7 and 36 6/7 weeks of gestation if it is not given earlier in pregnancy. The later recommendation is supported by an RCT (including 2827 infants) which showed that administration of betamethasone to these women significantly reduced the rate of neonatal respiratory complications [65]. We surveyed 449 obstetricians on gestational age beyond which antenatal steroids are not administered. The answer was 34 weeks by 294 obstetricians (65.5%), 37 weeks by 106 obstetricians (23.6%), 32 weeks by 42 obstetricians (9.3%), and 39 weeks by 7 obstetricians (1.6%). Therefore, we believe that administration of steroids for up to 37 weeks should be promoted in low resource setting where neonatal care of preterm babies is generally challenging. However, steroid usage may be contraindicated in some cases such as type I DM and sepsis.

Nevertheless, immediate delivery compared with expectant management after preterm pre-labor rupture of the membranes close to term (PPROMT trial) results were published in 2016. This RCT was conducted at 65 centers across 11 countries with a total of 1839 women randomized to either immediate intervention or expectant management. The study showed that neonates of patients assigned to immediate delivery had higher rates of respiratory distress (RR = 1.6, 95% CI = 1.1-2.3; *P* =  0.008), higher rates of mechanical ventilation (RR = 1.4, 95% CI = 1.0-1.8; *P* =  0.02) and longer admission to intensive care compared with neonates born to mothers in the expectant management group. However, women assigned to the expectant management group were at higher risks of antepartum or intrapartum hemorrhage (RR = 0.6, 95% CI = 0.4-0.9), intrapartum fever (RR = 0.4, 95% CI = 0.2-0.9), postpartum antibiotics (RR = 0.8, 95% CI = 0.7-1.0), and longer hospital stay. However, the risk of caesarean delivery was lower (RR = 1.4, 95% CI = 1.2-1.7) in the expectant group. Therefore, the study recommended expectant over immediate management at this gestational age [66]. A recent meta-analysis of 3 RCTs (2563 mothers, 2572 neonates) revealed comparable rates of the composite adverse neonatal outcomes between expectant and immediate managements. However, neonatal sepsis rates were 2.6% and 3.5%, respectively (RR = 0.74, 95% CI = 0.47-1.15). The risk of respiratory distress syndrome among neonates in the immediate delivery arm was higher (RR = 1.47, 95% CI = 1.10-1.97), and they were more likely to be admitted to the neonatal intensive care unit or special care nursery (RR = 1.17, 95% CI = 1.11-1.23). Immediate delivery was associated with lower risk of antepartum hemorrhage (RR = 0.57, 95% CI = 0.34-0.95) and IAI (RR = 0.21, 95% CI = 0.13-0.35), but higher risk of cesarean delivery (RR = 1.26, 95% CI = 1.08-1.47) [67].

Data from the Egyptian National Perinatal/Neonatal Mortality study in 2004 showed that neonatal mortality rate was 25 per 1000 live births; prematurity accounted for 39% of these cases. Perinatal mortality rate was 34 per 1000 births, 21% was attributed to prematurity. The mean gestational age of neonates diagnosed with early neonatal death (1-6 days) was 7.9 ± 1.1 months and 8.6 ± 0.8 months for late neonatal deaths (day 7 to 28). For stillbirths, the mean gestational age was 7.9 ± 1.3 months [68]. These numbers reflect that neonatal outcomes are less favorable among preterm neonates managed in low resource facilities compared to where the above studies were conducted. Therefore, we believe that expectant management should be adopted in low resource areas where management of neonatal complications is more challenging, highly expensive, and associated with poor prognosis compared to maternal complications, even among late preterm population. Gestational age at which the benefits of immediate delivery overweighs benefits of expectant management may be modified based on local perinatal data. Nevertheless, we recommend **expectant management up to 37 weeks of gestation** if PROM occurs beyond 34 weeks of gestation. RCOG has declared similar recommendations in 2019 based on these recent studies as well [69]. Regardless, delivery should be postponed at least 48 hours after administration of a course of prenatal steroids if maternal or fetal conditions are stable.

Administration of **antibiotics** should be similar to term PROM. However, women with unknown GBS status are treated as GBS positive and should be administered GBS prophylaxis. Latency antibiotics may be administered if expectant management to 37 weeks of gestation is elicited (see under ***PROM before 34 weeks of gestation***).

### PROM before 34 weeks of gestation

#### Expectant management

If diagnosis is made and both maternal and fetal status are reassuring, patient should be counselled on expectant management with **close monitoring** of signs of infection, abruption or labor which indicate termination of expectant management plan. As discussed earlier, hospitalization is the standard of care. Home care should not be offered as an alternative. If hospitalization is refused after informed counseling, safety criteria for possible home management as well as home monitoring of warning signs should be thoroughly discussed with the patient and the family. In both cases, **GBS swab** should be obtained for culture.

During hospital stay, monitoring of fetal heart rate and uterine contractions should be considered [70]. Frequency of monitoring can be modified based on cost and availability of tococardiography as well as clinical assessment and it should likely range from daily to twice weekly. Doppler fetal monitor can be used intermittently if resources are limited to rule out fetal tachycardia. However, it should not substitute intermittent tococardiography. Doppler fetal monitor can be used on daily basis if tococardiography cannot be available more than twice weekly. A recent meta-analysis of 12 studies (1744 fetuses) investigated the potential role of fetal thymus size in predicting neonatal outcomes. Pooled results from 5 of these studies showed increased rate of IAI among women at high risk (PROM and preterm labor) if fetal thymus measures small (OR = 16.0, 95% CI = 4.18-61.4). Furthermore, among women with PROM, the risk of neonatal sepsis increased (OR = 15.1, 95% 2.10-108) in the presence of small thymus, with a sensitivity and specificity of 95.0% (95% CI = 75.1-100) and 49.1% (95% CI = 41.2-57.0), respectively [71]. Analysis of these outcomes was limited by number of pooled patients (230 from 5 studies and 102 from 2 studies, respectively), significant heterogeneity, and wide range CI. Furthermore, standardization of thymus measurement, availability of trained sonographers, and establishment of clear cut-off measurements are barriers to implementing these results. In addition, application of these data in decision-making is still unclear, given the limited specificity of this sign and the lack of a feasible confirmatory test before active intervention is warranted.

The patient should be monitored for both vaginal bleeding and temperature. Sterile pads help to monitor the amount of bleeding and to differentiate stained amniotic fluid from active bleeding. She should be aware that fluid leak will likely recur intermittently and that the occurrence or the amount of fluid leak is unlikely to affect prognosis or treatment. This discussion helps to minimize patient anxiety as leakage continues. However, vaginal bleeding should be reported immediately. An episode of vaginal bleeding should be clinically assessed to rule out placental abruption and non-stress test should be conducted to monitor the fetus. IAI is monitored through clinical signs only; including fever, abdominal tenderness, purulent vaginal discharge, and fetal tachycardia, which should be evaluated and documented on daily basis. Although C-reactive protein (CRP) and white blood cell (WBC) count are popular in Middle-Eastern countries for infection monitoring and are performed on daily or twice weekly basis, no laboratory test has been found reliable for this indication. A recent meta-analysis of 13 studies was conducted to evaluate maternal laboratory predictors of chemical chorioamnionitis including maternal serum CRP and WBC. It concluded that both yield low sensitivity and specificity even when combined; 68.7% (95% CI = 58%-77%) and 77.1% (95% CI = 67%-84%), respectively. In 4 studies, maternal leukocytosis showed sensitivity of 51% (95% CI = 40%-62%) and specificity of 65% (95% CI = 50%-78%) [72]. Furthermore, obstetricians should be always reminded of the effect of antenatal steroids on maternal WBC count. Given patient inconvenience, costs and possibility of misguided premature interventions, we do not recommend these tests.

During the hospital stay, the patient should receive a single course of **corticosteroids** for enhancement of lung maturity, and **antibiotics** to protect against ascending infection. Also, **magnesium sulfate** is recommended as a neuroprotectant, to reduce the risk of cerebral palsy if labor is pending prior to 32 weeks of gestation. The number needed to treat to prevent one case of cerebral palsy is 63 (95% CI = 43-87) [73]. However, use of magnesium sulfate up to 33 weeks and 6 days is also recommended by RCOG [69].

Again, antibiotics should be wisely used. Antibiotics are indicated for GBS prophylaxis if labor is pending and GBS status is either positive or unknown. However, among expectantly managed women, antibiotics are administered as this has been found to prolong pregnancy duration and to decrease the risk of maternal and neonatal morbidities [1,12].

Two regimens of “latency” antibiotics were supported by 2 large RCTs. The National Institute of Child Health and Human Development Maternal-Fetal Medicine Units (NICHD-MFMU trial, 614 women) recommended the use of 48 hours of intravenous therapy (ampicillin 2 g and erythromycin 250 mg every 6 hours) followed by 5 days of oral medications (amoxicillin 250 mg and erythromycin 333 mg every 8 hours). This regimen was associated with pregnancy prolongation as well as reduction of neonatal morbidity (53% to 44%, *P* < 0.05), compared to placebo [74]. ORACLE I trial (4826 women) investigated oral antibiotic regimens and concluded that erythromycin 250 mg orally 4 times a day for 10 days is associated with pregnancy prolongation along with other neonatal benefits including less need for supplemental oxygen and lower rate of positive blood culture [75]. Based on this information, use of parenteral and oral penicillins and macrolides is recommended. Alternatively, prolonged erythromycin oral regimen for 10 days may be used specially in the presence of penicillin allergy or if hospitalization is refused by the patient. This regimen is currently supported by the RCOG [69].

It is important to emphasize that initial amniotic fluid volume should not influence patient care and decision making. A secondary analysis of NICHD-MFMU trial of 290 women, with singleton pregnancies at 24 to 32 weeks of gestation shows that low AFI and low MVP were present in only 67.2% and 46.9% of women, respectively. This finding was associated with shorter latency (*P* < 0.001), increased composite neonatal morbidity (*P* = 0.03), and increased respiratory distress syndrome (*P* ≤ 0.01), but not associated with increased risk of chorioamnionitis, neonatal sepsis (*P* = 0.85) nor pneumonia (*P* = 0.53) [76]. Therefore, the presence of oligohydramnios should not influence the decision of expectant management or administration of antibiotics. Similarly, follow-up of amniotic fluid volume is not necessary.

Of note, **tocolysis** is controversial and it should not be used to prolong pregnancy in the presence of uterine contractions because of increased risk of chorioamnionitis [77,78]. However, a physician may consider short term tocolysis if clinical situation justifies the risk such as to allow transfer to a tertiary care center or administration of steroids. Overall, the call for using tocolysis should be carefully made. If there is any suspicious for abruptio placenta, tocolysis is contraindicated and should perform immediate delivery.

#### Active management

Immediate delivery is indicated in the presence of non-reassuring fetal status, clinical evidence of infection, or significant placental abruption. Otherwise, expectant management should be conducted till 34 weeks of gestation. Although conservative management to 37 weeks seems to be an option as stated above, supportive studies recruited patients who had PROM between 34 and 37 weeks. Women with preterm PROM prior to 34 weeks would be exposed to a considerable risk of maternal and neonatal infection that likely overweighs the benefit of delayed delivery to the neonate. Given the fact that there is no perfect way to predict intrauterine infection, the risk of neonatal sepsis should be considered against the potential of adding unknown number of days to fetal maturity as data suggests increased infection risk with prolonged PROM [26].

Therefore, delivery at 34 weeks of gestation is a safe option. Nevertheless, the decision may be **individualized** depending on gestational age at preterm PROM and neonatology team input based on their local data. If neonatal outcome is considerably unfavorable at 34 weeks, extended conservation to 35-36 weeks is not unreasonable. It is important to directly connect the patient with a local neontologist to built-up realistic expectations. Hospitalization should be reinforced if the patient refuses earlier. However, data are insufficient to support this approach. A survey of 428 obstetricians showed that 298 (69.6%) endorsed expectant management until 37 weeks of gestation, 116 (27.1%) until 34 weeks of gestation, and 14 (3.3%) untill39 weeks of gestation.

### Pre-viable PROM

As discussed earlier, gestational age at viability is inconsistent. A recent large joint workshop in the USA defined periviable birth as delivery between 20 0/7 weeks to 25 6/7 weeks of gestation [79]. Although the definition of viability is less standardized in low-income countries, it lies between 25 0/7 to 28 0/7 weeks of gestation in most instances [9-11]. Pulmonary hypoplasia is the major risk factor of neonatal mortality [80]. The two major determinants of pulmonary hypoplasia are early gestational age of PROM and low residual amniotic fluid [81,82]. Pulmonary hypoplasia is rare if PROM occurs after 23-24 weeks of gestation, which is the critical gestational age for alveolar development [83]. This explains why the age of viability corresponds to this gestational age in developed countries. Nevertheless, lack of advanced care and equipment among most facilities in low resource areas is associated with high neonatal mortality due to severe respiratory distress syndrome or neonatal sepsis, hence, the discrepancy in gestational age of viability. Therefore, we recommend that, unless there are a definite local data, pregnancy between 24 and 28 weeks should be considered periviable and pregnant women who presents with Preterm PROM or preterm contractions should be **immediately transferred to a tertiary care center** with the highest neonatal intensive care for hospitalization if both the patient and the fetus are stable to transfer. Home care should not be an option [79]. Patient should be counseled on survival rates, morbidities, short term and long term outcomes of periviable birth through an involved neonatologist [79]. Finally, there is no current evidence to support routine cesarean section for delivery of women with periviable PROM unless obstetrically indicated; neonatal outcomes seem to be comparable to vaginal delivery [84,85].

However, prior to the age of viability, expectant management is not the standard of caregiven the high risk of maternal infection with prolonged latency. This risk is not justified given the poor neonatal outcome secondary to pulmonary hypoplasia specially in the presence of oligohydramnios or anhydramnios. A discussion should be conducted with the patient to allow a shared decision based on realistic expectations. However, patient decision should be supported and a plan that ensures patient safety should be discussed regardless of her decision.

If PROM occurs prior to the age of viability, hospitalization is not medically necessary as long as the patient is aware of warning signs of maternal infection and transfer to a tertiary center is feasible in a reasonable amount of time, ideally 15 to 30 minutes. However, hospitalization may be an acceptable option if patient safety at home cannot be ensured. Home management should be accompanied by a good follow-up plan; patients may feel motivated not to seek medical advice if they develop symptoms to avoid the decision of pregnancy termination. Sequences of maternal infection should be thoroughly discussed including the possibility of maternal sepsis and maternal mortality especially with delayed presentation. It is not unreasonable to schedule weekly visits to check maternal status and fetus viability. However, no intervention is proven to be beneficial at this stage including antenatal steroids, magnesium sulfate administration or treatment of GBS. Administration of latency antibiotics may be offered if the patient is interested in expectant management to prolong pregnancy and reduce the risk of neonatal infection. The earliest window when **antenatal steroids** may provide neonatal benefit is for babies delivered between 23th to 25th weeks of gestation. Neonatal benefits include reduction in neonatal mortality, intraventricular hemorrhage, periventricular leukomalacia, and necrotizing enterocolitis. [84,86]. No benefit is expected prior to this gestational age [87].

Less data are known regarding **magnesium sulfate** use for neuroprotection prior to age of viability. The 3 large clinical trials (conducted in Australia, France and USA), which highlights the protective role of magnesium sulfate against cerebral palsy, recruited women with a median (interquartile range) of 27 weeks and 3 days (25 5/7 weeks – 28 5/7 weeks), women whose gestational age ranges between 24 weeks to 32 weeks 6 days, and average gestational age of 28.3 ± 2.5 weeks, respectively [88-90]. Therefore, the standard of care is to administer magnesium sulfate after the age of viability. If a patient is managed conservatively and she reaches the gestational age of viability, she should be admitted to the hospital and managed like women with preterm PROM but with knowing that the prognosis is not the same (**Table 4**).

**Table 4:** MOGGE take home message: Treat

**At 37 weeks of gestation or beyond:**
• Delivery should be expedited.
• Antibiotics may be given to treat GBS if positive, or if IAI is clinically suspected. Digital pelvic examination should be minimized. Administration of prophylactic antibiotics is controversial; it may be considered if latency is longer than 12 hours
• Induction of labor via IV oxytocin seems to be superior to other options
• If Cesarean delivery is indicated, vaginal irrigation with povidone-iodine 1% is recommended to reduce the risk of endometritis and wound complications
**34 0/7 to 36 6/7 weeks of gestation:**
• Gestational age at delivery should be determined by local neonatal data. Expectant management may be planned up to 37 weeks of gestation if significantly unfavorable neonatal outcomes are anticipated with preterm labor
• Administration of antenatal steroids is recommended if not administered earlier in pregnancy
• Antibiotics can be given to treat GBS if positive or unknown. Latency antibiotics are also reasonable if expectant management is elicited
**Less than 34 weeks of gestation:**
• Hospitalization is the standard of care. Home care should not be offered as an alternative
• GBS swab should be obtained for culture
• During hospital stay, monitoring of fetal heart rate, uterine contractions, and clinical signs of IAI and placental abruption should be considered
• A single course of corticosteroids should be given for enhancement of lung maturity
• Magnesium sulfate is administered to reduce the risk of cerebral palsy if labor is pending prior to 32 weeks of gestation
• Antibiotics can be given for GBS prophylaxis) if labor is pending and GBS status is either positive or unknown
• Latency antibiotics should be given to prolong pregnancy and reduce the risk of neonatal morbidity
• Expectant management is reasonable up to 34 weeks of gestation. Further expectant management should be justified by consensus between obstetric and neonatology team based on their local data
• Immediate delivery is indicated in the presence of non-reassuring fetal status, clinical evidence of infection, or significant placenta abruption
**Pre-viable PROM:**
• Expectant management is not the standard of care. Hospitalization is not medically necessary
• A discussion should be conducted with the patient to allow a shared decision based on realistic expectations
• If expectant management is elicited, administration of latency antibiotics may be considered
• Administration of magnesium sulfate or antenatal steroids is not indicated
• Hospitalization is considered if pregnancy reaches gestational age of viability

MOGGE – Middle-East OBGYN Graduate Education. PROM – prelabor rupture of membranes, GBS – Group B streptococci, IAI – intra-amoniotic infection

## Additional material

Online Supplementary Document
