# Supplementary material for: Middle-East OBGYN Graduate Education (MOGGE) Foundation Practice Guidelines: Prelabor rupture of membranes; Practice guideline No. 01-O-19
Source: J Glob Health. 2020 Mar 30;10(1):010325. doi: 10.7189/jogh.10.010325 (PMC7125938; doi:10.7189/jogh.10.010325)
Supplement: Online Supplementary Document [file jogh-10-010325-s001.pdf]

Appendix S1. Summary of recommendations.

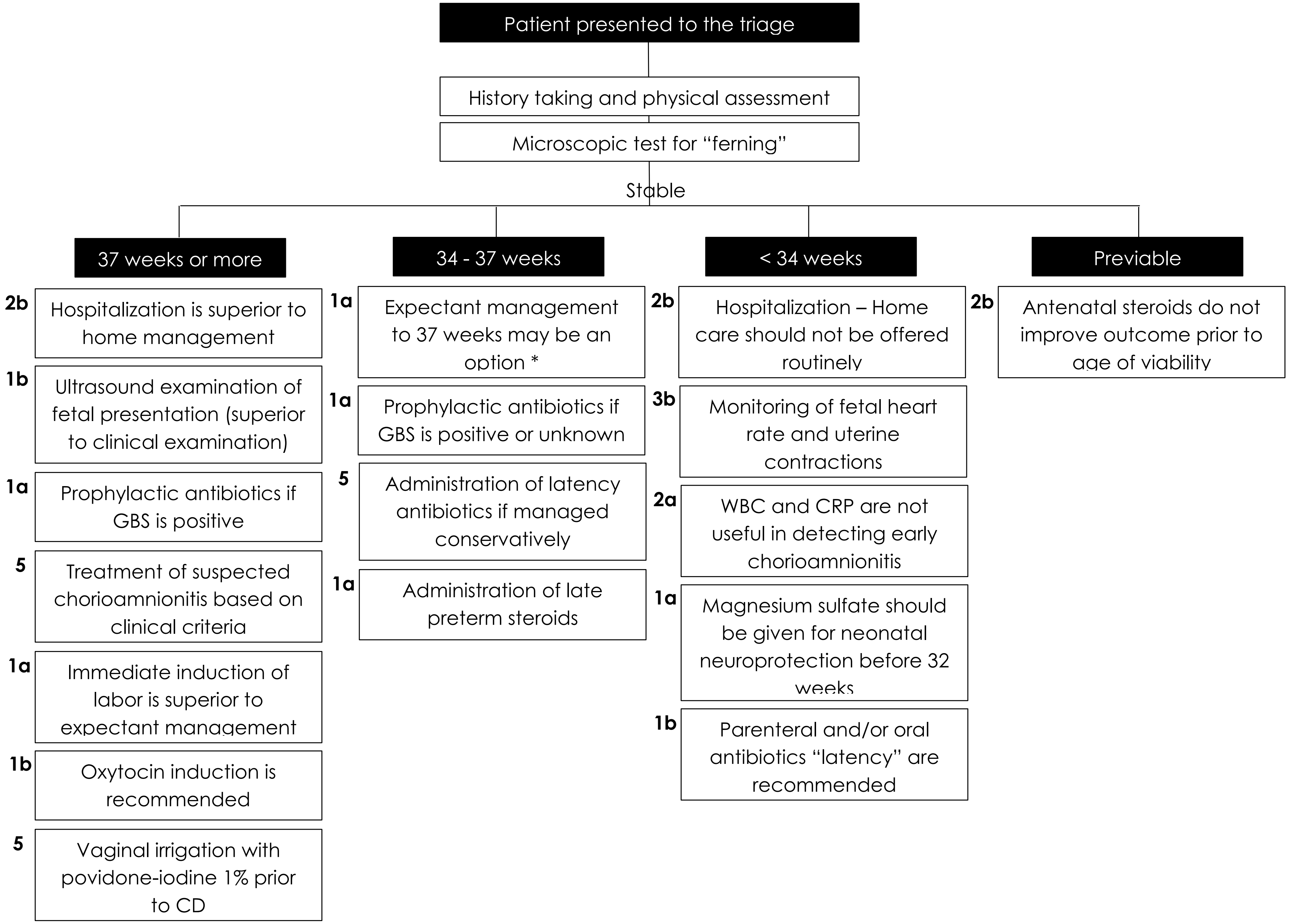

CD=Cesarean Delivery, WBC=White Blood Cell, CR*P*=C-Reactive Protein
